# Bioassessment of Cd and Pb at Multiple Growth Stages of Wheat Grown in Texturally Different Soils Using Diffusive Gradients in Thin Films and Traditional Extractants: A Comparative Study

**DOI:** 10.3390/plants13172445

**Published:** 2024-09-01

**Authors:** Hiba Shaghaleh, Sana Rana, Muhammad Zia-ur-Rehman, Muhammad Usman, Mujahid Ali, Hesham F. Alharby, Ali Majrashi, Amnah M. Alamri, Isam M. Abu Zeid, Yousef Alhaj Hamoud

**Affiliations:** 1 The Key Laboratory of Integrated Regulation and Resource Development on Shallow Lakes, Ministry of Education, College of Environment, Hohai University, Nanjing 210098, China; h-shaghaleh@hhu.edu.cn; 2 Institute of Soil and Environmental Sciences, University of Agriculture, Faisalabad 38000, Pakistanmusman07.mu@gmail.com (M.U.);; 3 Department of Biological Sciences, Faculty of Science, King Abdulaziz University, Jeddah 21589, Saudi Arabiaialmuan@kau.edu.sa (I.M.A.Z.); 4 Plant Biology Research Group, Department of Biological Sciences, Faculty of Science, King Abdulaziz University, Jeddah 21589, Saudi Arabia; 5 Department of Biology, College of Science, Taif University, P.O. Box 11099, Taif 21944, Saudi Arabia; 6 The National Key Laboratory of Water Disaster Prevention, College of Hydrology and Water Resources, Hohai University, Nanjing 210098, China

**Keywords:** agricultural productivity, texture class, aged contaminated, bioavailable fractions, DGT

## Abstract

The bioavailability of heavy metals in soil is a crucial factor in determining their potential uptake by plants and their subsequent entry into the food chain. Various methods, including traditional chemical extractants and the diffusive gradients in thin films (DGT) technique, are employed to assess this bioavailability. The bioavailability of heavy metals, particularly cadmium (Cd) and lead (Pb), is also influenced by soil texture and their concentrations in the soil solution. The primary objectives of this experiment were to compare and correlate the assessment of the Cd and Pb bioavailability using the DGT technique and traditional extractants across two soil textural classes: sandy clay loam (SCL) and clay loam (CL) at two contamination levels: aged contaminated (NC) and artificially contaminated (AC). The specific objectives included assessing the bioavailability of Cd and Pb at different growth stages of the wheat plant and correlating the DGT-based bioassessments of Cd and Pb with their concentrations in various plant parts at different growth stages. This study also compared the effectiveness of the DGT method and traditional extraction techniques in assessing the bioavailable fractions of Cd and Pb in soil. The regression analysis demonstrated strong positive correlations between the DGT method and various extraction methods. The results showed that the wheat plants grown in the AC soils exhibited lower root, shoot, and grain weights compared to those grown in the NC soils, indicating that metal contamination negatively impacts plant performance. The concentrations of Cd and Pb in the wheat tissues varied across different growth stages, with the highest levels observed during the grain filling (S3) and maturity (S4) stages. It is concluded that the in situ assessment of Cd and Pb though DGT was strongly and positively correlated with the Cd and Pb concentration in wheat plant parts at the maturity stage. A correlation and regression analysis of the DGT assessment and traditional extractants showed that the DGT method provides a reliable tool for assessing the bioavailability of Cd and Pb in soils and helped in developing sustainable soil management strategies to ensure the safety of agricultural products for human consumption.

## 1. Introduction

Soil contamination by pollutants has caused significant environmental problems [1]. This contamination results from the accumulation of contaminants such as fertilizers, heavy metals, pesticides, and inorganic chemicals from human activities, leading to toxic levels surpassing permissible limits [2,3]. In recent decades, heavy metal pollution has become a global issue due to industrial and agricultural activities [4]. Uncontrolled pollution releases have led to soil and water contamination, adversely affecting plant and animal life and making them unsuitable for human consumption [5]. Cadmium (Cd), chromium (Cr), mercury (Hg), copper (Cu), lead (Pb), and nickel (Ni) are among the heavy metals that exert the most significant negative impact on ecosystems [6,7,8]. Worldwide, approximately 35.9 million hectares of agricultural land are irrigated with urban wastewater, with 29.3 million hectares using minimally treated wastewater [9]. Metals like Cd and Pb persist in soils for extended periods as they are non-biodegradable, not easily eliminated through standard agricultural practices, and strongly bind to soil particles [10,11]. Assessing soil and water pollution levels around industrial areas has become crucial. Consequently, researchers and regulatory bodies are directing their efforts toward risk assessments of aquatic environments, soils, and subsequent food chain contamination to ensure safe food production [4,6,7,11].

The risk assessment of soil contamination depends on factors such as the contamination level, pollutant load, and potential ecological threats [12]. For a comprehensive analysis of various pollutants, it is essential to optimize sampling procedures and develop effective analytical techniques [13]. The methods used for sample collection and transfer can affect the distribution and form of chemical species before analysis [14]. Traditional methods yield the total extractable analytes, including physiologically inaccessible fractions [15,16,17]. Instead of focusing solely on the total analyte concentrations, assessing the labile fraction provides a better evaluation of the potential risks [4]. Evaluating metal bioavailability is crucial for determining the effectiveness of soil amendments in metal immobilization, highlighting the need for reliable analytical procedures in agricultural environmental science [18,19]. Plant absorption of heavy metals depends on soil bioavailability and plant tolerance, leading to varying toxicity levels when available heavy metal concentrations in the soil surpass their regulatory capacity [20].

Different approaches, including single and sequential chemical extractions, have been used to determine metal distribution in particle size fractions [7]. Metals in water from soil pores are the primary focus for assessing accessible metal fractions [19]. However, the accuracy of this method in predicting metal bioavailability and plant transfer is uncertain due to its dependence on soil preparation and phase separation [21]. Analytical challenges include low concentrations, saline matrices, contamination risks, and limited access to remote areas. Thus, there is a need for innovative, user-friendly, and robust tools for analyzing trace elements in ocean waters. The diffusive gradients in thin films (DGT) method is an analytical technique for in situ pollutant identification [8]. These passive samplers enable multi-element pre-accumulation in water, sediments, and soils [22]. They consist of a polyacrylamide hydrogel diffusing layer and a Chelex-100^®^ resin binding layer [23]. Initially tested with Zn, Cd, Cu, Ni, Fe, and Mn [24], subsequent experiments incorporated different functional materials for extended sampling periods [23]. Over nearly 30 years, numerous studies have advanced sampling methods and discovered new binding agents. Early geochemical and environmental studies focused on inorganic pollutants such as Zn, Cd, Cu, Ni, Fe, Mn, P, Pb, As, Se, V, and Sb [11,19,21,22,23,24].

In situ passive sampling techniques like DGT prevent speciation changes and contamination during sampling. They address challenges such as matrix interference and low concentrations and are user-friendly and efficient. By comparing the results of DGT and the total concentration, the DGT-accessible proportion of trace metals in seawater can be determined [11]. DGT units have a binding layer, diffusive gel, and filter membrane [11]. The binding resin captures metals quickly, creating minimal free metal concentrations at the resin–gel interface [24]. Only free metals accumulate at the binding layer, meaning metal complexes must dissociate before DGT uptake [25]. The primary objective of this experiment is to compare the effectiveness of the DGT technique with traditional chemical extractants in assessing the bioavailability of Cd and Pb in soil. This comparison will be conducted across two soil textural classes: sandy clay loam (SCL) and clay loam (CL), focusing on two contamination levels: aged contaminated (NC) and artificially contaminated (AC). Additionally, this study aims to correlate the results obtained from both assessment methods. The specific objectives include evaluating the bioavailability of Cd and Pb at different growth stages of wheat (seedling, tillering, grain filling, and maturity) to understand how metal uptake changes over time. This study also seeks to correlate the DGT-measured Cd and Pb bioavailability with their accumulation in various wheat plant parts (roots, shoots, and grains) at different growth stages, validating DGT as a reliable predictor of metal uptake in crops.

## 2. Results

### 2.1. Response of Wheat Plant Growth and Concentration of Cd and Pb in Plant Tissues

**Figure 1:**
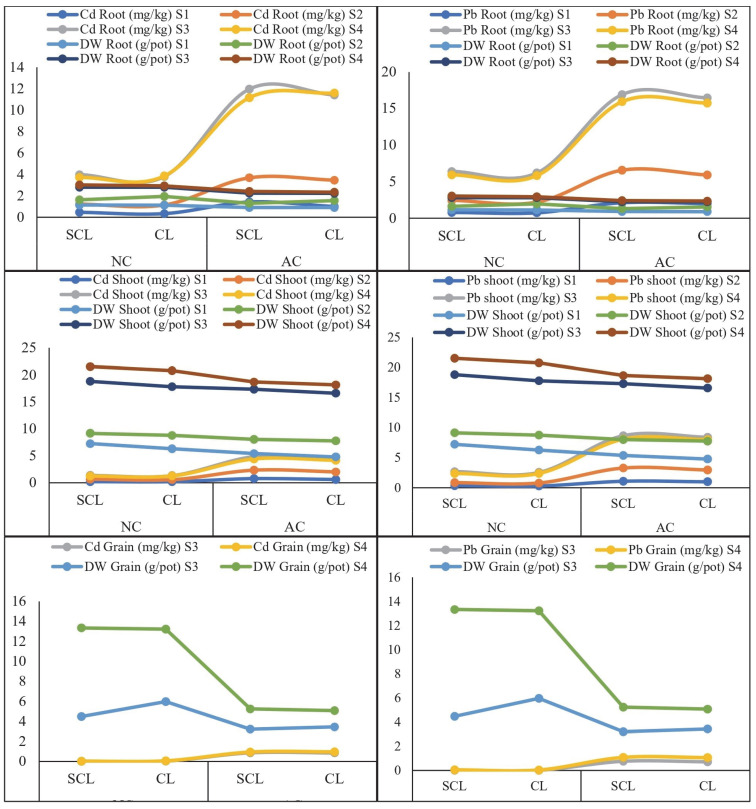
Wheat plant growth response and metal concentration in wheat plant tissues grown in texturally different contaminated soils. The different growth stages are represented as S1 (seedling), S2 (tillering), S3 (grain filling), and S4 (maturity), and DW represents dry weight. On X-axis: SCL; sandy clay loam, CL; clay loam, NC; aged contaminated, AC; artificially contaminated.

The experiment utilized soils with varying textures (SCL and CL) and different levels of metal contamination (NC and AC). Wheat plants were harvested at four distinct growth stages (S1, S2, S3, and S4). In the SCL-NC soil, the average root dry weights were 1.14 ± 0.03 g/pot at S1, 1.63 ± 0.04 g/pot at S2, 2.80 ± 0.05 g/pot at S3, and 3.03 ± 0.05 g/pot at S4. The trend observed in the root dry weight across these growth stages was S4 > S3 > S2 > S1. A similar trend was noted in the SCL-AC soil, although the root dry weights were lower compared to those in the NC soil. In the CL-NC soil, the maximum root dry weight occurred at S4, exceeding the weights observed at S3, S2, and S1, and was also higher than in the CL-AC soil, as depicted in Figure 1. In the case of the CL-AC soil, a similar trend was observed: at S4, there was a maximum dry weight of roots relative to that at S3, S2, and S1. For the shoot dry weight, the highest value was recorded at the S4 growth stage in the SCL-NC soil, reaching 21.53 ± 0.80 g/pot. The trend across the different growth stages followed the pattern S4 > S3 > S2 > S1 in the SCL-AC, CL-NC, and CL-AC soils. This indicates a consistent increase in the shoot dry weight as the plants progressed through the growth stages, as shown in Figure 1. Similarly, the highest wheat grain weight was recorded in the SCL-NC soil at the maturity stage (S4), measuring 13.34 ± 0.84 g/pot, and at the grain filling stage (S3), it was 4.47 ± 0.19 g/pot (Figure 1). Grain weight development primarily occurred during the S3 and S4 stages, with S4 showing a significantly greater response than S3 across all soil types, including the SCL-AC, CL-NC, and CL-AC soils. Overall, the CL soil demonstrated a better response in the root dry weight across both contamination levels (NC and AC).

The concentration of Cd and Pb in the wheat plant tissues varied across the different growth stages. In the SCL-NC soil, the maximum concentration of Cd in the roots and shoots was 3.99 ± 0.04 and 1.35 ± 0.05 mg/kg, respectively, at the grain filling stage (S3) and 0.02 mg/kg in grains at the maturity stage (S4), compared to the CL-NC soil. In the AC soil, the highest Cd concentrations in the roots and shoots were observed at the grain filling stage (S3), with values of 11.98 ± 1.04 and 4.84 ± 0.18 mg/kg, respectively, and 0.94 ± 0.05 mg/kg in the grains at the maturity stage (S4), which were higher in the SCL-AC soil than in the CL-AC soil (Figure 1). The trend for the Cd concentration in the roots and shoots followed the order S3 > S4 > S2 > S1 across all the soil types and contamination levels. For the Cd concentration in the grains, the trend was S4 > S3, while at S1 and S2, grain development had not yet occurred. Similarly, for the Pb concentration in the wheat plant tissues, the NC soil of both textural classes (SCL and CL) showed lower Pb concentrations in the roots, shoots, and grains compared to that in the AC soil of both textures. Among the different growth stages of the wheat plants, the grain filling stage (S3) exhibited the highest Pb concentrations in the roots and shoots, while the maturity stage (S4) showed the highest Pb concentration in the grains across all soil types (Figure 1). Among the different soil textural classes, the SCL soil exhibited the highest concentrations of both Cd and Pb compared to those in the CL soil. This pattern was consistent across all the growth stages of the wheat plants. In particular, the SCL soils showed a greater capacity to accumulate Cd and Pb in the plant tissues, resulting in higher concentrations in the roots, shoots, and grains. This indicates that the coarser texture of SCL soils may facilitate greater metal mobility and uptake by wheat plants, leading to more pronounced contamination in plant tissues relative to the finer-textured CL soils. The enhanced accumulation of these metals in the SCL soils highlights the importance of considering the soil texture when assessing the risk of heavy metal contamination in agricultural systems.

### 2.2. Uptake of Cd and Pb in Wheat Plant Tissues at Different Growth Stages in Different Types of Soils

**Figure 2:**
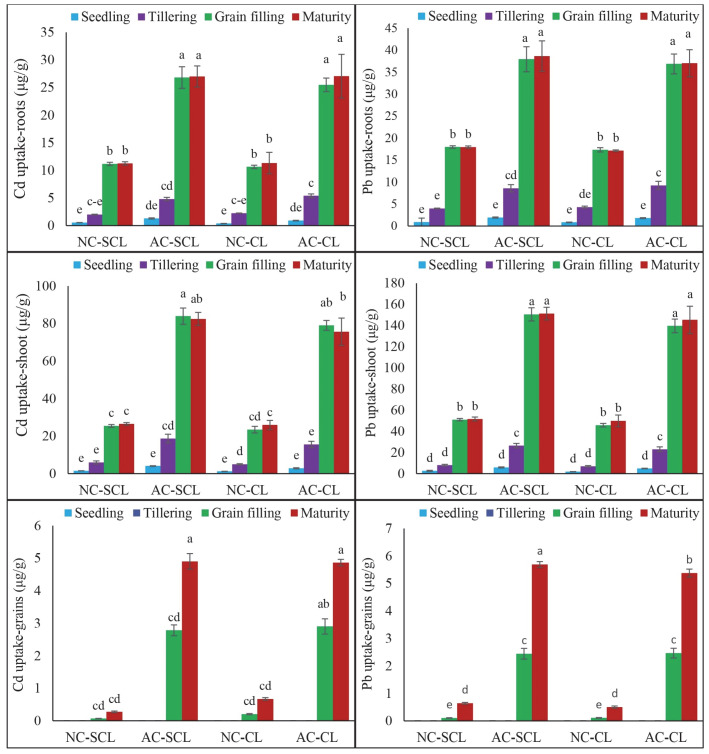
Uptake of Cd and Pb in wheat plant tissues grown in texturally different contaminated soils. The different growth stages are represented as S1 (seedling), S2 (tillering), S3 (grain filling), and S4 (maturity). On X-axis: SCL; sandy clay loam, CL; clay loam, NC; aged contaminated, AC; artificially contaminated. Bars of different colors showed the mean values, error bars indicating the standard error, and different lettering showing the significant difference (*p* < 0.05) of applied treatments.

The uptake of Cd and Pb in the wheat plant tissues across different growth stages and soil textures is depicted in Figure 2. The results show that the highest uptake of both Cd and Pb occurred in the roots, shoots, and grains at the maturity stage (S4), with a notable peak in Cd uptake in the shoots during the grain filling stage (S3). At the maturity stage, Cd uptake in the roots was significantly elevated, with values of 11.26 ± 0.30 μg/g DW in the SCL-NC soil, 27.01 ± 1.91 μg/g DW in the SCL-AC soil, 11.32 ± 1.95 μg/g DW in the CL-NC soil, and 27.05 ± 3.96 μg/g DW in the CL-AC soil. In contrast, Cd uptake in the roots was minimal during the seedling stage (S1) across all soil types, indicating a gradual increase in metal accumulation as the plants progressed through their growth stages. Cd uptake in the shoots exhibited some variation compared to the roots. In the non-contaminated (NC) soils, the grain filling stage (S3) was marked by the highest Cd uptake in both the SCL and CL textures. However, in the AC soils, the maturity stage (S4) demonstrated the highest Cd uptake in the shoots, reflecting the cumulative impact of prolonged exposure to Cd in these conditions. Cd uptake in the grains was consistently highest at the maturity stage (S4) across all soil types and contamination levels, underscoring the significant risk of Cd accumulation in edible plant parts under both NC and AC conditions. 

The Pb uptake in the wheat plants demonstrated a pronounced increase at the maturity stage across all soil types, with the most significant concentrations found in the shoots. Specifically, the Pb uptake in the shoots at the maturity stage reached 51.69 ± 1.84 μg/g DW in the SCL-NC soil, 151.27 ± 5.98 μg/g DW in the SCL-AC soil, 49.90 ± 5.50 μg/g DW in the CL-NC soil, and 145.34 ± 12.90 μg/g DW in the CL-AC soil. These results indicate that the SCL soil, especially under artificial contamination (AC), showed a higher propensity for Pb accumulation in the wheat shoots compared to that in the CL soil. The observed pattern suggests that as wheat plants progress towards maturity, their ability to uptake and retain Pb increases significantly, particularly in soils with higher contamination levels. This trend highlights the cumulative effect of prolonged exposure to Pb, where the metal becomes increasingly concentrated in plant tissues as they approach the end of their growth cycle. The Pb uptake in the grains followed a similar trend, with the highest levels of Pb accumulation also occurring at the maturity stage. Overall, the results emphasize that both the soil texture and contamination levels significantly influence Cd and Pb uptake in wheat, with sandy clay loam soils under artificial contamination conditions showing the highest levels of accumulation.

### 2.3. Translocation and Harvest Indices of Cd and Pb in Wheat Plants 

**Figure 3:**
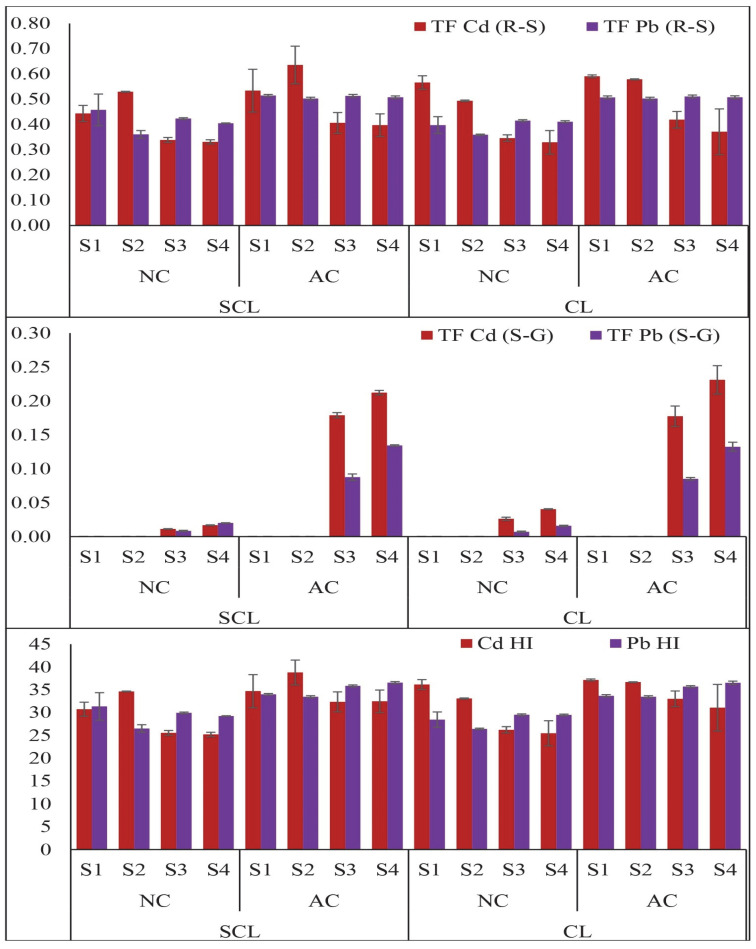
Translocation and harvest index of Cd and Pb in wheat plant tissues grown in texturally different contaminated soils. The different growth stages are represented as S1 (seedling), S2 (tillering), S3 (grain filling), and S4 (maturity). On X-axis: SCL; sandy clay loam, CL; clay loam, NC; aged contaminated, AC; artificially contaminated.

The translocation of Cd and Pb in the wheat plants was significantly influenced by the contamination levels, soil texture, and the plant’s growth stages, as shown in Figure 3. The translocation index of Cd from the roots to shoots was highest at the tillering stage (S2), reaching 0.53 in the SCL-NC soil, while the minimum was observed at S4 (0.33) < S3 (0.34) < S1 (0.44) in the SCL-NC soil. A similar trend was observed in the SCL-AC soil, where the translocation index followed the order S2 > S1 > S3 > S4. In contrast, the CL soils, under both contamination levels, exhibited a more effective translocation of Cd and Pb from the roots to shoots compared to that in the SCL soils. The translocation of Cd and Pb from the shoots to grains was also influenced by the soil texture and contamination levels. The maximum translocation factor of Cd from the shoots to grains was observed to be 0.02 in the SCL-NC soils and 0.21 in the SCL-AC soils. In the CL soils, the translocation factor was slightly higher, with values of 0.03 in the CL-NC soils and 0.23 in the CL-AC soils. A similar response was observed for Pb translocation from the root to shoot and shoot to grain. The translocation of Pb was at its maximum at the grain filling stage and at its minimum at the seedling stage. The contamination level also affected the translocation of Pb from the roots to shoots and shoots to grains. The soil texture influenced this factor, with the SCL soil showing increased translocation of Pb compared to that in the CL soil. The harvest index was also affected by all these factors, as depicted in Figure 3. Overall, these results highlight the complex interplay between soil texture, contamination levels, and growth stages in determining the translocation of heavy metals within wheat plants. The findings suggest that CL soils, due to their finer texture, may pose a greater risk for the movement of Cd and Pb from the soil to the edible parts of the plant, particularly under higher contamination levels. The harvest index was highest at the grain filling stage and lowest at the seedling stage across both contamination levels and soil textures.

### 2.4. Determination of Cd and Pb Concentration in Soil Using DGT and Single-Step Traditional Extraction Methods

**Figure 4:**
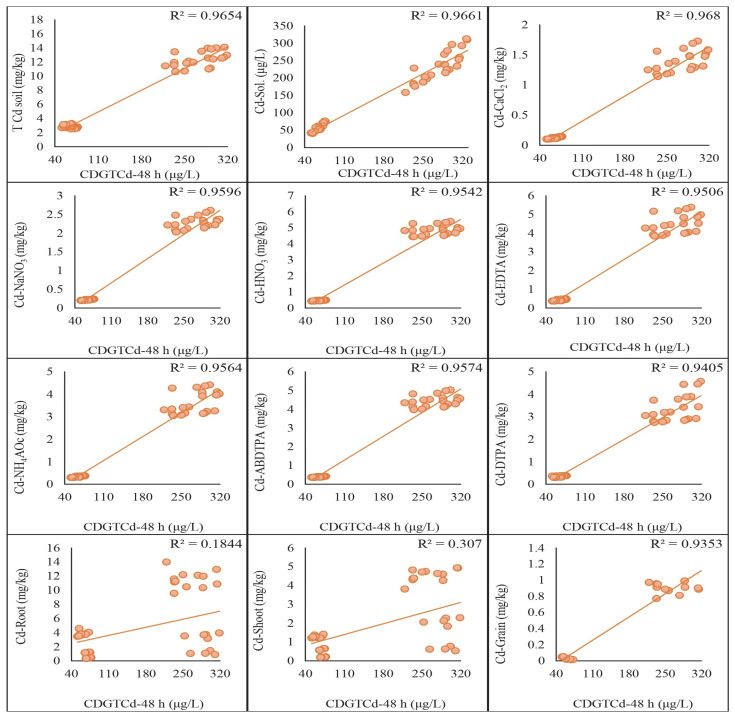
Regression analysis of Cd concentration extracted by extracts with CDGT at 48 h.

**Figure 5:**
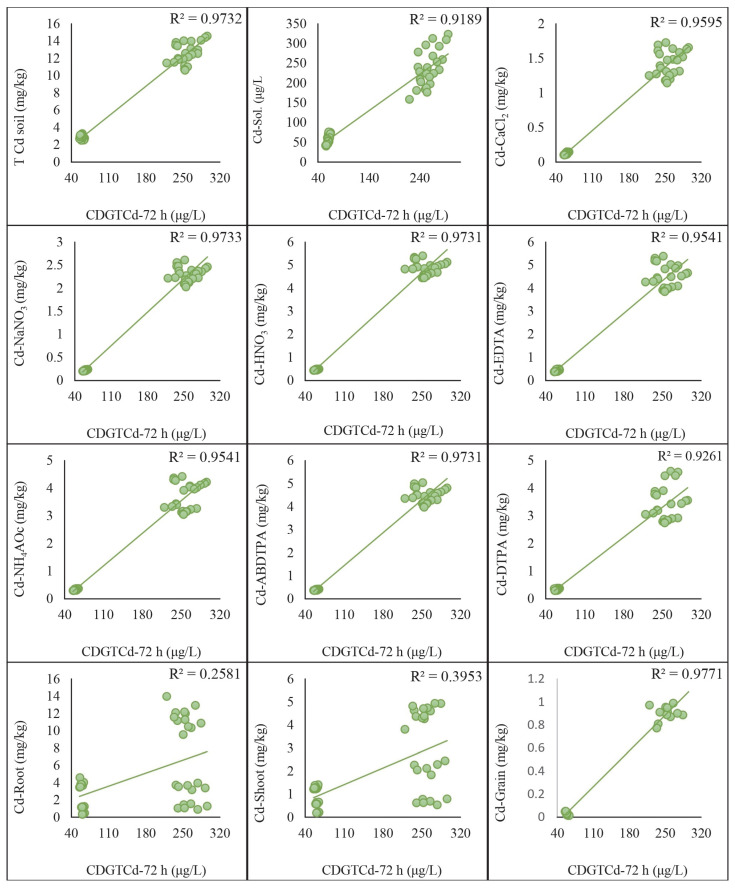
Regression analysis of Cd concentration extracted by extracts with CDGT at 72 h.

**Figure 6:**
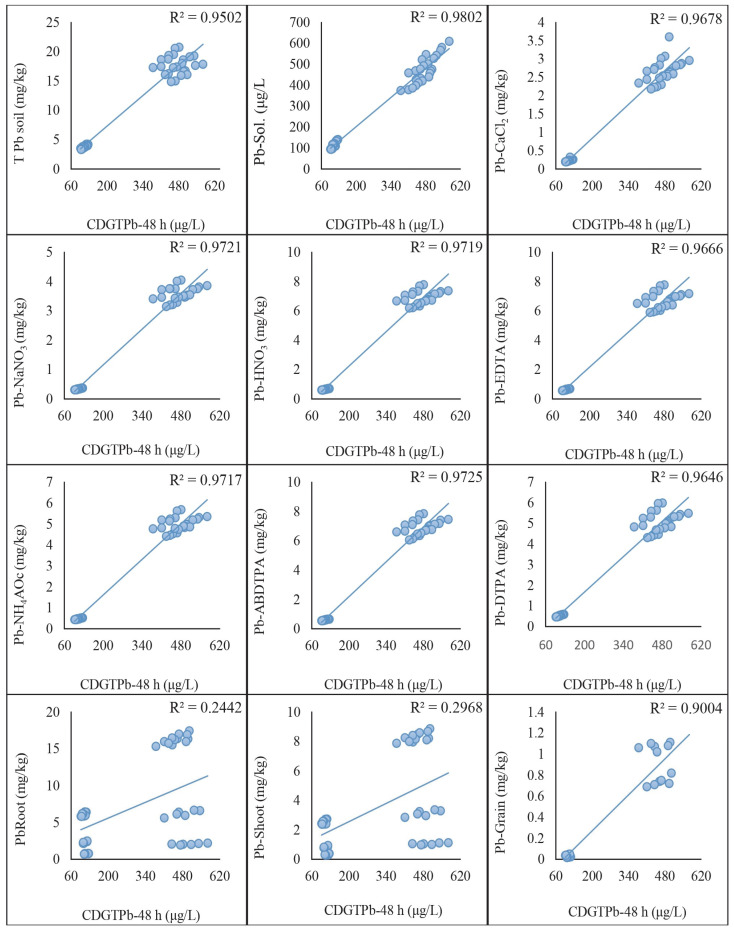
Regression analysis of Pb concentration extracted by extracts with CDGT at 48 h.

**Figure 7:**
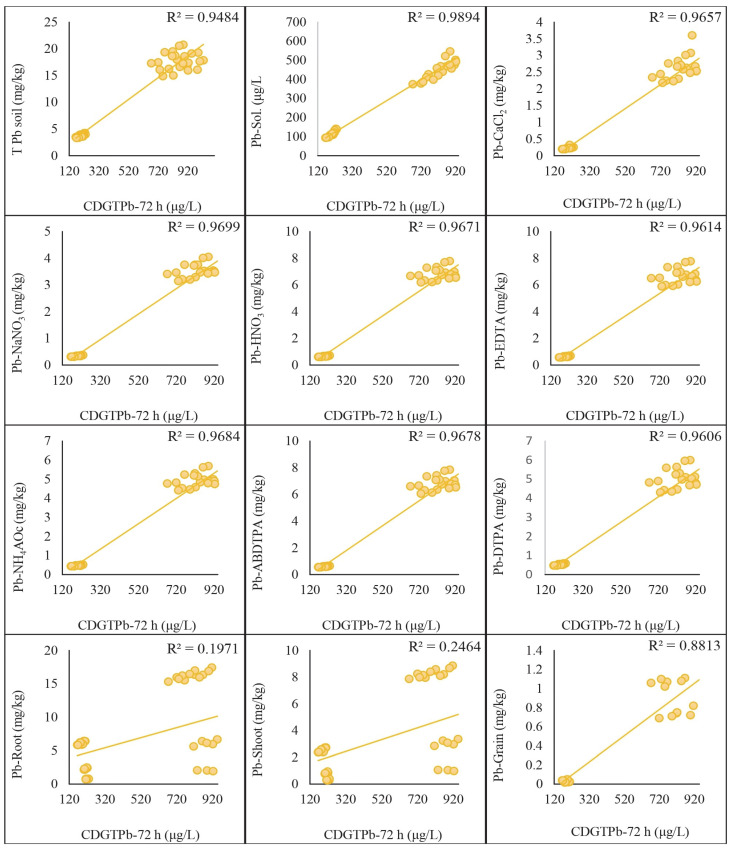
Regression analysis of Pb concentration extracted by extracts with CDGT at 72 h.

In this experiment, the concentration of Cd and Pb in soil was assessed using two methods: DGT and single-step traditional extraction methods (as shown in Figure 4, Figure 5, Figure 6 and Figure 7). The concentrations of Cd and Pb were measured in soil samples subjected to traditional extraction methods, while DGT devices were installed in each pot at specific growth stages for durations of 48 and 72 h. The results showed significant variations in the concentrations depending on the method used. Using the DGT method, the average concentration of Cd, calculated by CDGT, was found to be 76.17 ± 0.68, 73.48 ± 1.28, 70.82 ± 1.56, and 63.18 ± 3.45 μg/L at S1, S2, S3, and S4, respectively, when the DGT devices were exposed for 48 h in the SCL-NC soils. A similar trend was observed in the SCL-AC, CL-NC, and CL-AC soils for the concentration of Cd using DGT for 48 h. The highest concentration of Cd in the DGT devices at 48 h was found in Cd-spiked soil compared to aged contaminated soils. At 72 h, the CDGT values were 63.50 ± 2.04, 62.97 ± 2.22, 62.03 ± 1.53, and 59.47 ± 1.42 μg/L at S1, S2, S3, and S4, respectively, which were lower than the CDGT values after 48 h. The same trend was recorded in the SCL-AC, CL-NC, and CL-AC soils for the concentration of Cd measured using DGT for 72 h across all growth stages of the wheat plants. The highest concentration of Cd in the DGT devices after 72 h was also found in the Cd-spiked soil compared to the aged contaminated soils. Using the DGT method, the average concentration of Pb was found to be 124.60 ± 1.90, 119.70 ± 1.00, 114.40 ± 3.01, and 112.11 ± 1.86 μg/L at S1, S2, S3, and S4, respectively, after 48 h of exposure in the SCL-NC soil. Similar trends were observed in the SCL-AC, CL-NC, and CL-AC soils. The highest Pb concentration at 48 h was found in the Pb-spiked soil compared to the aged contaminated soils. At 72 h, the CDGT values increased to 227.00 ± 1.32, 219.50 ± 1.00, 205.80 ± 3.83, and 193.70 ± 2.57 μg/L at S1, S2, S3, and S4, respectively. This trend was consistent across the SCL-AC, CL-NC, and CL-AC soils, with the highest Pb concentrations again found in the Pb-spiked soil. The assessment of the Cd and Pb concentration through the DGT devices showed that maximum concentration of Cd and Pb was observed at the seedling stage (S1) > tillering stage (S2) > grain filling stage (S3) > maturity stage (S4).

With the single-step extraction methods, the assessment of Cd showed an increasing trend, as follows: HNO_3_ (0.50 mg/kg) > EDTA (0.48 mg/kg) > ABDTPA (0.42 mg/kg) > DTPA (0.38 mg/kg) > NH_4_AOc (0.37 mg/kg) > NaNO_3_ (0.24 mg/kg) > CaCl_2_ (0.15 mg/kg) in the SCL-NC soil at the seedling growth stage. A similar trend was recorded in both textural classes of the aged contaminated and artificially contaminated soils. The growth stages showed a decreasing trend for the extraction of Cd with traditional extractants, which was S4 < S3 < S2 < S1, as indicated in Figure 4, Figure 5, Figure 6 and Figure 7. For Pb, traditional extractants such as CaCl_2_, NaNO_3_, HNO_3_, NH_4_AOc, ABDTPA, DTPA, and EDTA showed a similar trend to the extraction of Cd at different growth stages in all types of soil. The highest Pb concentrations were observed with HNO_3_, followed by EDTA, ABDTPA, DTPA, NH_4_AOc, NaNO_3_, and CaCl_2_.

### 2.5. Regression Analysis of DGT-Assessed Cd and Pb with Traditional Extraction Methods

The regression analysis was conducted to compare the concentrations of Cd and Pb assessed by the DGT method with those obtained using traditional extraction methods. The results indicated significant correlations between the two methods, highlighting their comparative effectiveness in assessing metal concentrations in soil. A strong positive correlation was found between the Cd concentrations measured by the DGT method and those obtained using HNO_3_, EDTA, ABDTPA, DTPA, NH_4_Ac, NaNO_3_, and CaCl_2_ extraction methods. The correlation coefficients (R^2^) for each extraction method with CDGT 48 h were as follows: HNO_3_: 0.95, EDTA: 0.95, ABDTPA: 0.96, DTPA: 0.94, NH_4_Ac: 0.96, NaNO_3_: 0.96, CaCl_2_: 0.97. These were achieved by keeping the growth stages and soil types constant (Figure 4). Similarly, the correlation coefficients (R^2^) derived from the regression analysis of CDGT at 72 h with extractants were as follows: HNO_3_: 0.97, EDTA: 0.95, ABDTPA: 0.97, DTPA: 0.93, NH_4_Ac: 0.95, NaNO_3_: 0.97, CaCl_2_: 0.96. These are given in Figure 5. Similarly, the Pb concentrations assessed by the DGT method showed strong positive correlations with those obtained using the HNO_3_, EDTA, ABDTPA, DTPA, NH_4_Ac, NaNO_3_, and CaCl_2_ extraction methods. The correlation coefficients (R^2^) for each extraction method with CDGT-48 were as follows: HNO_3_: 0.97, EDTA: 0.97, ABDTPA: 0.97, DTPA: 0.97, NH_4_Ac: 0.97, NaNO_3_: 0.97, CaCl_2_: 0.97 (Figure 6). The regression analysis of CDGT at 72 h with extractants gave the following correlation coefficients (R^2^): HNO_3_: 0.97, EDTA: 0.96, ABDTPA: 0.97, DTPA: 0.96, NH_4_Ac: 0.97, NaNO_3_: 0.97, CaCl_2_: 0.967. These are provided in Figure 7. The regression analysis demonstrates that the DGT method is a reliable alternative to traditional extraction methods for assessing the concentrations of bioavailable Cd and Pb in soil. The strong correlations suggest that both methods can be effectively used for soil contamination studies, providing consistent and comparable results.

### 2.6. Regression Analysis of DGT-Assessed Cd and Pb with Wheat Plant Tissue Concentrations

The regression analysis was performed to compare the concentrations of Cd and Pb assessed by the DGT method with those measured in different tissues of wheat plants. The results indicated significant correlations, demonstrating the effectiveness of the DGT method in predicting metal uptake by plants. A strong positive correlation was observed between the Cd concentrations measured by the DGT method and those found in the wheat plant tissues (roots, shoots, and grains). The correlation coefficients (R^2^) for each plant tissue with CDGT-48 were as follows: roots: 0.18, shoots: 0.31, grains: 0.94. These high correlation coefficients suggest that the DGT method accurately reflects the bioavailable Cd that the wheat plants take up. The following regression equations were also derived from the analysis: Cd-roots = (0.0163 × CdCDGT_48 h) + 1.8042, Cd-shoot = (0.0081 × CdCDGT_48 h + 0.5022), and Cd-grain = (0.0041 × CdCDGT_48 h − 0.198). These predict the relationship between CDGT-48 and the Cd concentration in the roots, shoots and grains of the wheat plants. A regression analysis between CDGT-72 and the Cd concentration in the roots, shoots, and grains was also performed and equations were derived to predict the Cd concentration, which were Cd-Roots = (0.0215 × CdCDGT_72 h) + 1.2232, Cd-Shoot = (0.0103 × CdCDGT_72 h) + 0.2825, and Cd-Grain = (0.0044 × CdCDGT_72 h − 0.2254). Similarly, the Pb concentrations assessed by the DGT method showed strong positive correlations with the Pb concentrations in the wheat plant tissues. The regression prediction models for the Pb concentration in the wheat plant tissues and CDGT of Pb at 48 h are given as Pb-root = (0.0153 Pb × CDGT_48 h) + 2.5443, Pb-shoot = (0.0088 × PbCDGT_48 h) + 0.7931, and Pb-grain = (0.0024 × PbCDGT_48 h) − 0.217 (Figure 6). The same was performed for the regression analysis of the CDGT of Pb at 72 h with the Pb concentration in the wheat plant tissues, which gives the prediction model as Pb-root = (0.0075 × PbCDGT_72 h + 3.0129), Pb-shoot = (0.0044 × PbCDGT_72 h) + 1.031, and Pb-grain = (0.0014 × PbCDGT_72 h − 0.2083) (Figure 7). These results indicate that the DGT method effectively predicts the bioavailable Cd and Pb that are accumulated in the wheat plants. The regression analysis demonstrates that the DGT method is a reliable tool for predicting the concentrations of bioavailable Cd and Pb that wheat plants take up. The strong correlations between the DGT-assessed metal concentrations and those in the plant tissues support the use of DGT for assessing the potential risks of metal uptake in crops.

### 2.7. Correlation Analysis of Studied Parameters

**Figure 8:**
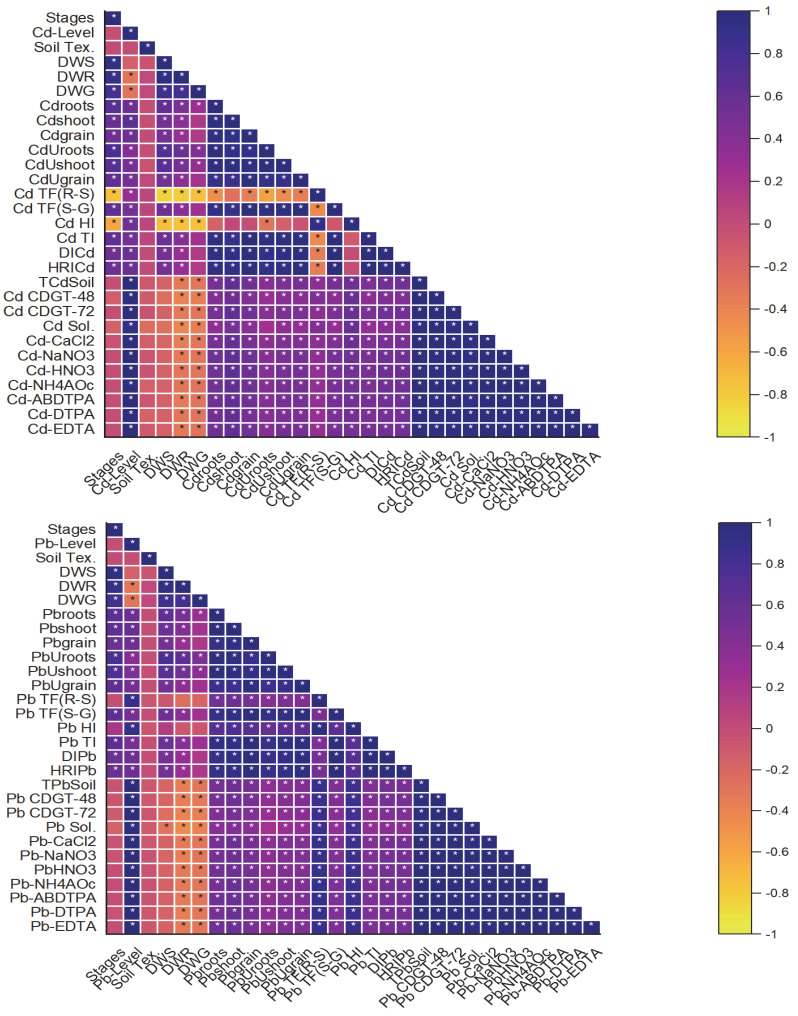
Correlation analysis of studied parameters. Without stars indicated in the figures showing the non-significant (*p* > 0.05) correlation among the studied parameters while tars show the parameters were significantly (*p* < 0.05) correlated to each other.

The correlation analysis examined the relationships between various studied parameters, shedding light on their interdependencies and implications. Strong positive correlations were observed between the Cd and Pb concentrations in the soil samples. Both the Cd and Pb concentrations in the wheat plant tissues (roots, shoots, and grains) showed significant positive correlations with their corresponding soil concentrations. The growth stages of the wheat plants (seedling, tillering, grain filling, and maturity) exhibited varying correlations with the Cd and Pb uptake, with the grain filling stage showing the strongest correlation. The soil texture (SCL vs. CL) and contamination levels (aged contaminated vs. artificially contaminated) influenced the correlations between the soil metal concentrations and uptake by wheat plants, with higher correlations observed in the soils with higher contamination levels and specific textures (Figure 8). The correlation analysis highlights the complex interactions between the Cd and Pb concentrations in soil, their uptake by wheat plants at different growth stages, and the influence of soil properties. These findings show the importance of considering multiple factors in assessing the risks associated with metal contamination in agricultural environments.

## 3. Discussion

### 3.1. Wheat Plant Growth Response and Concentration of Cd and Pb in Plant Tissues

This study demonstrates that the soil texture (SCL and CL) and metal contamination (NC and AC) significantly impact wheat growth and development. Lower root, shoot, and grain weights in AC soils indicate that metal contamination negatively affects plant performance, consistent with studies reporting reduced crop growth in contaminated soils due to nutrient imbalance, oxidative stress, and reduced uptake of water and nutrients [26,27,28,29]. The growth stage is also crucial, with root, shoot, and grain weights increasing from early stages (S1 and S2) to later stages (S3 and S4), reflecting higher resource allocation as plants mature. This aligns with the concept of ontogenetic drift, where plants adjust resource allocation with age [30]. These findings have significant implications for sustainable agriculture, especially in metal-contaminated areas [31]. Understanding how soil properties, metal contamination, and growth stages interact can help develop strategies for sustainable wheat production in diverse environments [27,28].

This study shows significant variation in Cd and Pb concentrations in wheat tissues across growth stages and soil types. In the AC soils, the metal concentrations were highest in the roots, followed by the shoots and grains, influenced by soil properties affecting metal uptake. During the grain filling stage (S3), the roots and shoots had the highest Cd and Pb levels, while the grains peaked at maturity (S4). This indicates increased metal translocation to the grains during these stages, highlighting the importance of understanding these responses to reduce contamination in food. Cadmium levels in the area pose a risk, with mean concentrations in the seed, stem, and root at 0.29, 0.62, and 0.95 mg/kg, respectively, exceeding the EU’s 0.20 mg/kg limit for wheat grains [32]. Wheat accumulates Cd mostly in the roots and stems, showing resistance to transfer [33]. Cd accumulation increases from early tillering to maturity due to soil-to-plant transfer over time [30]. High Pb levels in the roots are due to high soil Pb content and properties like pH, organic matter, and cation exchange capacity, affecting its bioavailability [34,35,36]. Wheat restricts Pb translocation to the edible parts, limiting contamination.

### 3.2. Uptake of Cd and Pb in Wheat Plant Tissues

The data show that the uptake of Cd and Pb was highest in the roots at the maturity stage across all the soil types and contamination levels. This suggests that the roots are the primary site of heavy metal accumulation in wheat plants, particularly during the later stages of growth. Similar findings have been reported in other studies, where roots were found to accumulate higher concentrations of heavy metals compared to other plant tissues [28,37,38]. The Cd uptake in the shoots was highest at the grain filling stage in the NC soils, while in the AC soils, the maturity stage showed the highest Cd uptake. This indicates that the soil contamination level and growth stage may influence the shoot’s response to Cd uptake. Previous studies have also reported variations in heavy metal uptake by shoots based on the growth stage and soil contamination levels [39]. The uptake of Cd and Pb in the grains was highest at the maturity stage across all soil types and contamination levels. This is a concerning finding, as the accumulation of these heavy metals in the edible parts of the wheat plant can pose a potential risk to human health if the grains are consumed [39,40]. The translocation of heavy metals from roots to grains is a well-documented phenomenon, and several studies have reported the presence of Cd and Pb in wheat grains grown in contaminated soils [41,42,43,44].

### 3.3. Translocation and Harvest Indices of Cd and Pb in Wheat Plants at Different Growth Stages in Various Contaminated Soils

The results demonstrate that the contamination level, soil texture, and growth stage significantly influence the translocation of Cd and Pb in wheat plants. The findings indicate that Cd and Pb translocation from roots to shoots and from shoots to grains is minimal during the seedling stage but reaches a maximum at the grain filling stage across all soil types and contamination levels. This growth stage-dependent pattern of heavy metal translocation is consistent with the findings of previous studies. Rizwan et al. [45] reported that cereal crops exhibit a higher capacity to restrict the transport of Cd to the grains during the early growth stages, while the translocation increases as the plants progress towards maturity. This is likely due to the increased demand for nutrients and water during the grain filling stage, which can facilitate the uptake and translocation of heavy metals within the plant [30,31,46]. Similarly, Fang et al. [7] observed that the accumulation of Pb in wheat grains was significantly higher at the grain filling stage compared to that in earlier growth stages. The results also highlight the role of soil texture and contamination level in modulating heavy metal translocation in wheat plants. The higher Cd and Pb translocation observed in the SCL soils compared to the CL soils may be attributed to differences in soil properties, such as pH, organic matter content, and cation exchange capacity, which can influence the bioavailability and mobility of these metals [26,27]. Similarly, the increased translocation of Cd and Pb in the AC soils compared to the NC soils suggests that wheat plants have a limited ability to restrict the uptake and transport of these metals when grown in highly polluted environments. This finding is consistent with the results of previously published work, which observed that the accumulation of heavy metals in wheat grains is directly proportional to their availability in the soil [29,47].

### 3.4. Assessment of Cd and Pb Concentration in Soil Using DGT and Single-Step Traditional Extraction Methods

The findings presented in the given information provide valuable insights into the assessment of Cd and Pb concentrations in soil using two different methods: the DGT technique and single-step traditional extraction methods. The results highlight the variations in Cd and Pb concentrations depending on the method used and the growth stage of the wheat plants. The DGT technique was used to measure the concentrations of Cd and Pb in soil samples at different growth stages of the wheat plants. The results showed that the average CDGT was highest at the seedling stage (S1) and decreased with subsequent growth stages in both aged contaminated and artificially contaminated soils. This trend was consistent across both soil textures (sandy clay loam and clay loam) and exposure durations (48 and 72 h). Similar findings have been reported in other studies where the DGT technique was used to assess the bioavailability of heavy metals in soils [11,30,48]. The highest concentrations of Cd and Pb were observed in the artificially contaminated soils compared to the aged contaminated soils, which is expected due to the higher metal concentrations in the spiked soils [49]. The DGT technique provides a measure of the labile metal fraction, which is considered to be more bioavailable and potentially toxic to plants and soil organisms [22,23,50]. 

The assessment of Cd and Pb concentrations using single-step traditional extraction methods showed varying results depending on the extractant used. The extractants followed a decreasing trend in the order HNO_3_ > EDTA > ABDTPA > DTPA > NH_4_AOc > NaNO_3_ > CaCl_2_ for both Cd and Pb. This trend was consistent across the different soil types and growth stages. The growth stages showed a decreasing trend for the extraction of Cd and Pb with traditional extractants, with the highest concentrations observed at the seedling stage (S1) and decreasing with subsequent growth stages. This could be attributed to the changes in soil properties and the uptake of metals by the growing wheat plants over time. Single-step extraction methods provide information about the total extractable metal content in the soil, which may not necessarily represent the bioavailable fraction [49,51]. However, these methods are widely used for routine soil analysis and provide a general indication of the metal status in the soil.

### 3.5. Comparative Effectiveness of DGT and Traditional Extraction Methods through Regression Analysis 

The regression analysis conducted in this study demonstrates the comparative effectiveness of the diffusive gradients in the DGT method and traditional extraction methods in assessing the concentrations of Cd and Pb in soil. The strong positive correlations observed between the DGT method and various extraction methods, such as HNO_3_, EDTA, ABDTPA, DTPA, NH_4_Ac, NaNO_3_, and CaCl_2_, highlight the reliability and consistency of both approaches in quantifying the bioavailable fractions of these heavy metals in soil. The high correlation coefficients (R^2^) ranging from 0.926 to 0.973 for Cd and 0.961 to 0.973 for Pb across different extraction methods and DGT sampling times (48 and 72 h) suggest that the DGT method can provide comparable results to traditional extraction techniques. This finding is consistent with the results of previous studies that have reported the effectiveness of the DGT method in predicting metal bioavailability and plant uptake [48,52,53]. The DGT method offers several advantages over traditional extraction techniques. Unlike single-point extractions, the DGT method provides a time-integrated measure of metal availability, which is more representative of the dynamic nature of metal interactions in the soil–plant system [54,55]. Additionally, the DGT method mimics the uptake of metals by plant roots, making it a more ecologically relevant approach for assessing metal bioavailability [56,57,58]. Furthermore, the DGT method is less labour-intensive and requires smaller sample sizes compared to traditional extraction methods. It also avoids potential artefacts associated with soil disturbance and sample preparation, which can alter metal speciation and availability [59].

### 3.6. Regression Analysis of DGT-Assessed Cd and Pb with Wheat Plant Tissue Concentrations

The regression analysis presented in the information provides strong evidence for the effectiveness of the DGT method in predicting the uptake of Cd and Pb by wheat plants. The findings demonstrate the significant correlations between the metal concentrations measured by the DGT method and those found in the different tissues of the wheat plants. These findings are consistent with those of other studies that have reported the ability of the DGT technique to predict metal uptake by plants [54,60]. The use of DGT for predicting Pb uptake by plants has been demonstrated in several studies [48,56,61]. The high correlation coefficients and the derived regression equations demonstrate the effectiveness of the DGT method in predicting the bioavailable fractions of Cd and Pb that the wheat plants take up. This is consistent with findings from other studies that have reported the ability of the DGT technique to accurately reflect the bioavailable metal concentrations in soil and their subsequent uptake by plants [62]. The DGT method provides a reliable measure of the bioavailable fraction of Cd and Pb in the soil, which is more relevant for assessing the potential uptake by plants and the associated risks to the food chain [10]. The regression equations derived from the analysis can be used to develop predictive models that estimate the concentrations of Cd and Pb in different wheat plant tissues based on the DGT-measured values. This can be a valuable tool for risk assessment and decision-making in agricultural systems.

## 4. Materials and Methods

### 4.1. Soil Collection and Characterization

Soil from two wheat-producing districts in Punjab irrigated with raw city effluents for 30 years was collected from the top 15 cm and transported to the University of Agriculture Faisalabad. After air-drying, grinding, and sieving through a 2 mm mesh, a sample was taken for pre-experiment analysis. Physicochemical properties, including pH and organic matter (determined using the Walkley-Black method), cation exchange capacity (determined by ammonium acetate extraction), and the total and bioavailable concentrations of Cd and Pb (determined by aqua regia and AB-DTPA extraction methods, respectively), were analyzed. Soluble cations and anions were assessed by titration and physical properties like soil saturation percentage and texture were measured using gravimetric and hydrometric methods. Results are detailed in Table 1.

### 4.2. Spiking of Soil and Pot Filling

The prepared soil was divided into two groups collected from both districts: one was aged contaminated, and the other spiked with Cd and Pb at a rate of 15 mg/kg. The soil was spiked with Cd and Pb using 3CdSO_4_.8H_2_O and Pb_2_SO_4_, respectively, which were sprayed onto the soil and mixed thoroughly. The spiked soils were then incubated for two months in the shade, and distilled water was added to equilibrate the metal distribution. After the incubation period, the spiked soil was air-dried, ground, and sieved again. The spiked soils of different textures and the aged contaminated soils of different textures were filled into pots at a rate of 10 kg per pot. Two textural classes of soils with two types of contamination were used in the experiment.

### 4.3. Cultivation of Wheat and Experimentation

Wheat seeds (variety: Akbar-2019) were collected from the university’s seed bank, and five seeds were sown per pot. The recommended dose of fertilizers (NPK at 120:90:60 kg/ha) was applied using urea, diammonium phosphate, and sulphate of potash, respectively. The full dose of these fertilizers was applied just before sowing the seeds. Irrigation was performed with distilled water, and the pots had no drainage due to lining with a polythene sheet. After germination, the pots containing sandy clay loam (SCL) and clay loam (CL) soils with aged contaminated levels of Cd and Pb (NC) and artificially contaminated levels of Cd and Pb (AC) were divided into four sub-groups based on growth stages. These growth stages were: seedling stage (15 days after sowing; S1), tillering stage (35 days after sowing; S2), grain filling stage (70 days after sowing; S3), and maturity (120 days after sowing; S4). The experiment was designed as a completely randomized design with three replications. It was based on two soil textural classes (SCL and CL), two contamination levels (NC and AC), and four growth stages (S1, S2, S3, and S4).

### 4.4. Arrangement and Deployment of DGT Devices

The DGT devices, along with Chelex and diffusive gels, were imported from the United Kingdom (UK). The Chelex gel sheet was placed on a marble slab, washed with deionized water, and cleaned with ethanol. It was then cut into 2.5 cm diameter pieces by pressing and twisting the cutter for a clean cut. The filter membrane was moistened with ultra-pure water. The assembly process involved placing the Chelex gel resin-side up on the base, followed by the diffusive gel and then the filter membrane. The cap was secured by applying horizontal force to ensure a tight fit. The assembled DGT devices were wetted with water and sealed in clear plastic bags, then stored at 4 °C in a refrigerator to maintain proper membrane moisture. When deploying the DGT devices, they were removed from the refrigerator without opening the sealed bags, ensuring moisture retention. The devices were then deployed in pots at specific harvesting times for each treatment. Before deployment, the soil was moistened with deionized water. Each pot received six DGT units, which were pressed and rotated to ensure good soil contact with the membrane filter. A temperature sensor was installed to monitor average temperatures during deployment. The DGT units remained in wheat-growing pots containing 10 kg of homogenized contaminated soil, ensuring adequate moisture. Deployment times of 48 and 72 h were specified for each growth stage of wheat plants. At the end of each deployment period, DGT units were carefully removed without touching the exposed window end and rinsed with a water stream from a wash bottle. The units were then stored in new, clean plastic bags with appropriate moisture for subsequent analysis.

### 4.5. Assessment of Cd and Pb Adsorption on DGT Devices

After the homogenization period (48 and 72 h), the washed DGT devices were used to quantify metal adsorption. Labelled tubes were weighed before and after adding 2 mL of 1 M HNO_3_ for 24 h. The device cap was removed by inserting a screwdriver into the groove, and the filter membrane and diffusive gel were peeled away. The Chelex gel was gently washed with ultra-pure water and placed on filter paper to dry. The digested resin gel solution was then analyzed using an atomic absorption spectrometer (AAS). The concentration of Cd and Pb in the DGT (C_DGT) was calculated using the equation modified from Zhang and Davison [63]:

CDGT = MΔg/DAt

Here, M is the accumulated Cd or Pb (μg), Δg is the diffusive layer thickness (cm), D is the diffusion coefficient of Cd or Pb (cm^2^/s), A is the DGT window area (cm^2^), and t is the deployment time (s).

### 4.6. Soil Solution and Single-Step Traditional Extraction Methods

The soil solution concentrations of Cd and Pb were measured using traditional centrifugation with distilled water. After harvesting wheat plants from each pot at various growth stages, soil samples were collected and subjected to single-step extraction methods outlined in Table 2. Following extraction and filtration, the filtrate was analyzed for Cd and Pb concentrations using AAS.

### 4.7. Harvesting and Determination of Cd and Pb

After reaching specific growth stages, as mentioned in Section 2.3, wheat plants were harvested, and their biomass was determined. Samples were dried at 65 ± 5 °C until constant weight, then ground into powder using a stainless steel grinder. The powdered samples were digested with HNO_3_ and HClO_4_ acids at a ratio of 3:1 on a hot plate at 200 °C until the clear solution appeared in the digestion flask. Post-digestion, the samples were diluted with distilled water in volumetric flaks (volumetric flask of 10 mL for NC and 25 mL volumetric flask for AC), filtered through Whatman 42 filter paper, and analyzed using Atomic Absorption Spectroscopy (AAS) after running the standards of different concentration.

### 4.8. Secondary Parameter Calculations

The translocation factor for root to shoot was calculated by dividing the concentration of Cd and Pb in the shoot to root; for the shoot to grains, it was calculated as the metal concentration in the grain divided by metal concentration in the shoot.

### 4.9. Statistical Analysis

Stepwise multiple linear regressions were used to analyze the relationships between CDGT-48 and CDGT-72 with traditional extractants and plant tissues for Cd and Pb using MSO Excel 2019. The normality of data distribution was assessed using Kolmogorov–Smirnov and Shapiro–Wilk tests, all analyzed with IBM SPSS Statistics version 23.0. The test identified treatment differences at the 5% probability level, with each treatment replicated three times by using SPSS. Correlation among the studied parameters was performed by Origin 2022.

## 5. Conclusions

Based on the results, this study demonstrates that the soil texture and contamination levels profoundly affect the growth and development of wheat plants. Specifically, the SCL soils, especially under AC, exhibited higher metal accumulation compared to the CL soils. The wheat plants showed varying growth responses across the different soil types and contamination levels, with the maximum root and shoot dry weights observed at the maturity stage in the SCL-NC soil. The grain weights also peaked in the SCL-NC soils at maturity. Notably, the highest concentrations of Cd and Pb were found in the roots, shoots, and grains at maturity, with the SCL soils showing greater metal accumulation than the CL soils.

Additionally, the comparison between the DGT technique and traditional chemical extractants (e.g., HNO_3_, EDTA, ABDTPA, DTPA, NH_4_Ac, NaNO_3_, CaCl_2_) revealed that DGT offers a more precise assessment of the metal bioavailability. DGT effectively captured the labile fractions of Cd and Pb across both soil textures and contamination levels, validating its reliability as a predictive tool over conventional methods. This study also established a strong correlation between the DGT-measured bioavailability of Cd and Pb and their accumulation in the wheat roots, shoots, and grains. These findings underscore the necessity for improved soil management practices to address metal contamination, particularly in sandy clay loam soils. Understanding DGT’s effectiveness in assessing metal bioavailability provides valuable insights for managing metal uptake in crops and mitigating risks to food safety and environmental health. Overall, this study shows the importance of understanding heavy metal dynamics in wheat–soil systems, advocating for DGT as a robust tool in assessing the metal bioavailability and plant uptake.

## Figures and Tables

**Table 1:** Pre-experiment characteristics of soil.

Parameters	Units	Values	
Sand	%	54.44	41.89
Silt	%	22.01	26.56
Clay	%	23.55	31.55
Soil Texture		Sandy clay loam	Clay loam
Saturation percentage	%	33.00	35.00
Soil-saturated paste pH		7.78	7.69
Electrical conductivity	dS/m	4.78	4.99
Cation exchange capacity	cmol_c_/kg	12.00	15.00
Sodium adsorption ratio	(mmol/L)^1/2^	10.45	11.55
Total Cd	mg/kg	2.75	3.10
Total Pb	mg/kg	4.18	3.78
ABDTPA Cd	mg/kg	0.45	0.42
ABDTPA Pb	mg/kg	0.71	0.63
Organic matter	%	0.78	0.83

**Table 2:** Single-step traditional extraction methods.

Extraction Method	Soil Weight (g)	Solution Composition	Solution Volume (mL)	Shaking Time	References
AB-DTPA	10	1 N NH_4_HCO_3_ + 0.005 M DTPA	20	15 min	[64]
0.005 M DTPA	10	0.005 M DTPA + 0.01 M TEA + 0.01 M CaCl_2_	20	2 h	[65]
0.05 M EDTA	2	0.05 M EDTA, pH 7.0	20	1 h	[66]
0.01 M CaCl_2_	2	0.01 M CaCl_2_	20	3 h	[67]
0.1 M NaNO_3_	8	0.1 M NaNO_3_	20	2 h	[68]
0.43 M HNO_3_	2	0.43 M HNO_3_	20	2 h	[69]
1 N NH_4_.AOc	2	1 N NH_4_.AOc	20	2 h	[70]

## Data Availability

Data will be made available on request.
